# Predicted resting metabolic rate and prognosis in patients with ischemic stroke

**DOI:** 10.1002/brb3.2911

**Published:** 2023-02-07

**Authors:** Xiaoyu Lin, Aichun Cheng, Yuesong Pan, Mengxing Wang, Xia Meng, Yongjun Wang

**Affiliations:** ^1^ Department of Neurology, Beijing Tiantan Hospital Capital Medical University Beijing China; ^2^ China National Clinical Research Center for Neurological Diseases Beijing China; ^3^ Research Unit of Artificial Intelligence in Cerebrovascular Disease Chinese Academy of Medical Sciences Beijing China; ^4^ Center for Excellence in Brain Science and Intelligence Technology Chinese Academy of Sciences Beijing China

**Keywords:** basal metabolism, ischemic stroke, prognosis

## Abstract

**Purpose:**

Resting metabolic rate (RMR) could represent metabolic health status. This study aims to examine the association of the predicted RMR with 1‐year poor functional outcome and all‐cause mortality in patients with ischemic stroke as a proxy of metabolic profile.

**Methods:**

A total of 15,166 patients with ischemic stroke or transient ischemic attack (TIA) from the Third China National Stroke Registry (CNSR‐III) were enrolled in this study. The Harris–Benedict equation based on sex, age, weight, and height was used to predict RMR. The primary endpoints were poor functional outcome defined as ≥3 modified Rankin Scale (mRS) score and all‐cause mortality within 1 year. The association between predicted RMR and prognosis was assessed by multivariable regression analysis. Besides that, subgroup analysis of age, sex, and body mass index (BMI) with predicted RMR was also performed.

**Results:**

12.85% (1657) individuals had poor functional outcome and 2.87% (380) died of whatever causes within 1 year. An inverse association was found between predicted RMR with poor functional outcome and all‐cause mortality. Compared to the lowest quartile, the highest quartile was significantly associated with lower risk of poor functional outcome (adjusted odds ratio [OR], 0.43 [95% confidence interval (CI) 0.33–0.56]) and all‐cause mortality (adjusted hazard ratio [HR], 0.44 [95% CI 0.28–0.71]). No significant interaction was between predicted RMR and specified subgroup.

**Conclusions:**

Predicted RMR by the Harris–Benedict equation seems to be an independent protective predictor of poor functional outcome and all‐cause mortality after ischemic stroke as a metabolic proxy.

## Graphical abstract

Predicted resting metabolic rate by equation has a reversely significant association with all‐cause mortality and poor functional outcome after is hemic stroke that indicates it seems to be a protective predictor of poor outcome of ischemic stroke as a metabolic proxy.

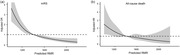

## 1 INTRODUCTION

Ischemic stroke is the leading cause of physical disability and causes enormous economic burdens (Wang et al., 2020). It is imperative to recognize the related markers and factors associated with poststroke results in arrange to direct clinical stroke administration way better. Emerging evidence suggested that metabolic related factors, such as obesity, insulin resistance, type 2 diabetes mellitus, have a great implication for stroke incidence as well as its prognosis (Lau et al., 2019; Rodríguez‐Castro et al., 2019; Yang et al., 2021).

Resting Metabolic Rate (RMR), defined as the daily energy required to ensure life‐sustained functions in resting status, takes up 60–70% of the total daily energy demands (Zampino et al., 2020). Age, height, weight, sex, genetic variation, and physiology all are responsible for RMR. Previous studies indicated that RMR may represent the metabolic health status as a marker of whole body metabolism level. For example, a recent report from the European Prospective Investigation into Cancer and Nutrition (EPIC) showed that the higher basal metabolic rate estimated by WHO/FAO/UNU equation was associated with a greater risk of specific cancers (Kliemann et al., 2020). Another supportive example was that the higher metabolic rate assessed using indirect calorimetry an older adult had, the greater burden of multimorbidity he/she might have (Fabbri et al., 2015). That indicated that the RMR may be an indicator for prognosis after ischemic stroke as a proxy of metabolic profiles same as for cancer or natural death (Kliemann et al., 2020; Ruggiero et al., 2008). However, the effects of RMR on poststroke prognosis have not been investigated in depth. It is worth investigating the relationship between them in terms of better prognosis management of ischemic stroke.

This study aims to assess the association between predicted RMR as a marker of metabolic profile and the prognosis of ischemic stroke based on the Third China National Stroke Registry (CNSR‐III) database (Wang et al., 2019). Owing the initial design of the CNSR‐III database did not include the RMR variable measured by indirect calorimetry, we used the predicted RMR by the Harris–Benedict equation as an alternative.

## 2 METHODS

### 2.1 Study design

This study was based on baseline and follow‐up data from the CNSR‐III study. The rationale, design, and baseline participant characteristics of the CNSR‐III had been described previously (Wang et al., 2019). Briefly, the CNSR‐III, a nationwide prospective registry for patients presented to hospitals with acute ischemic cerebrovascular events, recruited 15,166 patients with ischemic stroke or transient ischemic attack (TIA) within 7 days from the onset of symptoms between August 2015 and March 2018 in China. Ischemic stroke was diagnosed according to the World Health Organization criteria and confirmed by Magnetic Resonance Imaging or brain Computed Tomography. In this study, participants were excluded if they presented with one of the following: (1) age < 30 or > 80 years; (2) body mass index (BMI) < 16 or > 40 kg/m^2^; (3) cancer at recruitment; (4) discharge diagnosis of TIA.

### 2.2 Standard protocol approvals, registrations, and patient consents

The study was approved by the ethics committees of Beijing Tiantan Hospital (IRB approval number: KY2015‐001‐01) and all participating centers. Written informed consents were signed by all participants or their legal proxies before enrolling in the study.

## 3 DATA COLLECTION

Baseline data on demographic characteristics, medical and medication history, laboratory findings as well as clinical situations were collected by trained research coordinators at admission. Height and weight were measured according to standardized methods. Height was measured to the nearest 0.1 cm and weight was measured to the nearest 0.1 kg wearing only light underwear. Self‐reported height and weight were collected if the information were impossible to be collected. BMI was calculated as weight (kg) divided by height square (m^2^). The functional state was evaluated using modified Rankin Scale (mRS) score. Stroke severity at admission was assessed by trained neurologists according to the National Institutes of Health Stroke Scale (NIHSS) score. The etiology of ischemic stroke was classified according to the TOAST (Trial of ORG 10172 in Acute Stroke Treatment) criteria. Fasting blood samples were collected within 24 h of admission and were frozen in cryotube at −80°C refrigerator. Samples were sent to the central laboratory in Beijing Tiantan Hospital and measured by laboratory technicians blinded to the baseline data.

### 3.1 Assessment of the predicted resting metabolic rate

Harris–Benedict equation derived early in 1919 is one of the most frequently used to predict RMR in clinical application (Harris & Benedict, 1918). This method calculates RMR using sex‐specific equations and is also based on the participant's age, weight, and height. Accumulating evidence indicated that predicted RMR by the Harris–Benedict equation was associated well with that by indirect calorimetry in nonobesity, healthy participants and even more so in those with obesity (Bendavid et al., 2021). We chose the Harris–Benedict equation to assess RMR. Meanwhile, RMR was also calculated by using the other three common equations, Oxford, Mifflin St Jeor, and WHO/FAO/UNU for each individual for comparison according to Nathalie et al (Supplemental Table S1) (Energy & Protein Requirements, 1985; Henry, 2005; Kliemann et al., 2020; Mifflin et al., 1990).

### 3.2 Outcome and follow‐up

Follow‐up time started from the day of enrollment in the registry project. Patients were followed up for 1 year after ischemic stroke by trained research coordinators over the telephone. Information on functional status and all‐cause death was collected. Each case death was confirmed according to a death certificate from the attended hospital or the local citizen registry. The primary study endpoints included poor functional outcome, defined as functional dependency based on mRS score of 3 to 5, and all‐cause mortality resulting from the index event or other causes in 1‐year follow‐up.

### 3.3 Statistical analysis

The predicted RMR at baseline was calculated by the Harris–Benedict equation and also was assessed using Mifflin St Jeor, WHO/FAO/UNU, and Oxford equations. Pearson's correlation coefficients between RMR defined by the Harris–Benedict equation and other equations were derived.

Patients were dived into four groups according to predicted RMR quartiles and we chose the lowest quartile or first quartile as a reference. Continuous variables were expressed as median (interquartile range, IQR). Categorical variables were presented as proportions. Kruskal–Wallis test was used for comparisons of continuous variables. Categorical variables were compared with the χ^2^ statistics or Fisher's exact test as appropriate. Predicted RMR was analyzed according to a quartile or a continuous variable. The relationship between predicted RMR with the 1‐year poor functional outcome and all‐cause mortality was investigated using multivariable logistic regression analysis (odds ratio [OR] and 95% confidence intervals [CI]) and Cox proportional hazards regression analysis (hazard ratio [HR] and 95% CI), respectively. Additionally, the association between ordinary mRS (7 levels with 0–6 score) and predicted RMR was also assessed with logistic regression analysis. We constructed two adjusted models to evaluate the primary endpoints: sex adjusted model and multivariable adjusted model. The latter model incorporated the following variables: sex, BMI, current smoking, hypertension, dyslipidemia, diabetes mellitus, prior stroke, prior atrial fibrillation (AF), prior coronary heart disease (CHD), NIHSS and mRS at admission, TOAST subtype, recombinant tissue plasminogen activator intravenous thrombolysis (rt‐PA IVT) treatment, antithrombotic drugs, and lipid‐lowering drugs. To vigorously assess the relationship between multivariable adjusted primary endpoints and predicted RMR on a continuous scale, we depicted restricted cubic splines with five knots (at the 5th, 27.5th, 50th, 72.5th, and 95th centiles). Kaplan–Meier curves associated with predicted RMR were compared using log‐rank tests by an original unadjusted model. In addition, we performed prespecified subgroup analysis to evaluate the effects of sex (man; woman), age (<60; ≥60), BMI (<25; ≥25) on predicted RMR. In sensitivity analysis, we exclude the population with mRS of 4 to 5 at admission and conducted univariable and multivariable analysis in patients with the mRS of 0 to 3.

A two‐sided *p* value<.05 was considered significant. All of the analyses were performed with SAS software, version 9.4 (SAS Institute, Inc., Cary, NC)

## 4 RESULTS

In CNSR‐III, a total of 15,166 ischemic stroke or TIA patients were finally eligible and had complete information at baseline. Therein, 769 individuals not according with age of 30–80 years old limit, 45 individuals out of BMI of 16 to 40 limit, 109 individuals with reported cancer at recruitment, and 1020 individuals diagnosed TIA when discharged. Therefore, 1943 cases were excluded and the rest of 13,223 out of 15,166 individuals (87%) of ischemic stroke were eligible for analysis with median age of 62 years, 69.4% male, median predicted RMR of 1408.0 kcal/day.

Patients with higher predicted RMR reported greater BMI and a tendency of current smoking. But patients with lower predicted RMR were more likely to suffer from cardiovascular and cerebrovascular diseases. Moreover, those who were in the lowest quartile were more likely to have a higher initial NIHSS score compared to other quartiles (Table 1). Predicted RMR calculated applying the Harris–Benedict equation was significantly correlated (*r* ≥ .92) with those derived from the Mifflin St Jeor, WHO/FAO/UNU, and Oxford equations (Supplemental Table S2).

**TABLE 1:** Baseline characteristics of patients with ischemic stroke according to predicted RMR

		Resting metabolic rate (RMR) (kcal/d)				
Variables	Overall	Q 1(< 1270.9)	Q 2(1270.9–1408.0)	Q 3(1408.0–1552.8)	Q 4(>1552.8)	*p* Value
RMR, median (IQR)	1408.0 (1270.9–1552.8)					
Age, median (IQR)	62 (54–69)	68 (63–74)	63 (57–69)	61 (54–67)	53 (47–61)	<.0001
Male, *n* (%)	9174 (69.4)	854 (25.9)	2005 (60.6)	3039 (92.0)	3276 (99.1)	<.0001
BMI, median (IQR)	24.5 (22.7–26.6)	22.5 (20.6–24.2)	24.0 (22.5–26.1)	24.5 (23.4–26.0)	26.7 (25.3–28.4)	<.0001
Current smoking, *n* (%)	4367 (33.0)	426 (12.9)	874 (26.4)	1388 (42.0)	1679 (50.8)	<.0001
hs‐CRP, median (IQR)	1.77 (0.82–4.63)	1.92 (0.84–5.46)	1.74 (0.8–4.63)	1.68 (0.81–4.66)	1.73 (0.86–4.04)	.03
Medical history, *n* (%)						
Diabetes mellitus	3108 (23.5)	769 (23.3)	806 (24.3)	768 (23.2)	765 (23.1)	.63
dyslipidemia	1017 (7.7)	198 (6.0)	237 (7.2)	267 (8.1)	315 (9.5)	<.0001
Hypertension	8312 (62.9)	2050 (62.2)	2065 (62.4)	2030 (61.4)	2167 (65.6)	.0026
stroke	2921 (22.1)	732 (22.2)	734 (22.2)	784 (23.7)	671 (20.3)	.01
AF	818 (6.2)	290 (8.8)	205 (6.2)	184 (5.6)	139 (4.2)	<.0001
CHD	1336 (10.1)	412 (12.5)	339 (10.2)	340 (10.3)	245 (7.4)	<.0001
Num. of the chronic diseases, median (IQR)^a^	1 (1–2)	1 (1–2)	1 (1–2)	1 (1–2)	1 (1–2)	.33
Medication history, *n* (%)						
Lipid‐lowering drugs	1380 (10.4)	366 (11.1)	333 (10.1)	387 (11.7)	294 (8.9)	.001
Antithrombotic drugs	2276 (17.2)	578 (17.5)	591 (17.9)	597(18.1)	510 (15.4)	.02
mRS at admission, *n* (%)						.05
0–1	12027 (91.0)	2998 (90.9)	2981 (90.0)	3026 (91.6)	3022 (91.4)	
2–3	887 (6.7)	212 (6.4)	238 (7.2)	215 (6.5)	222 (6.7)	
4–5	304 (2.3)	88 (2.7)	92 (2.8)	63 (1.9)	61 (1.9)	
Initial NIHSS, median (IQR)	3 (2–6)	4 (2–7)	3 (2–6)	3 (1–6)	3 (1–6)	<.0001
TOAST subtype, *n* (%)						<.0001
LAD	3405 (25.8)	859 (26.1)	857 (25.9)	866 (26.2)	823 (24.9)	
SVO	761 (5.8)	268 (8.1)	199 (6.0)	161 (4.9)	133 (4.0)	
CE	2999 (22.7)	654 (19.8)	754 (22.8)	769 (23.3)	822 (24.9)	
Others^b^	6053 (45.8)	1517 (46.0)	1501 (45.3)	1508 (45.6)	1527 (46.2)	
rt‐PA IVT, *n* (%)	1439 (10.9)	393 (11.9)	368 (11.1)	325 (9.8)	353 (10.7)	.05

^a^ Includes diabetes mellitus, dyslipidemia, hypertension, stroke, atrial fibrillation, and coronary heart disease.

^b^ Includes other determined etiology and undetermined etiology.

Abbreviations: AF, atrial fibrillation; BMI, body mass index; CE, cardioembolism; CHD, coronary heart disease; hs‐CRP, high‐sensitive C‐reactive protein; LAD, large‐artery atherosclerosis; NIHSS, National Institutes of Health Stroke Scale; RMR, resting metabolic rate; rt‐PA IVT, recombinant tissue plasminogen activator intravenous thrombolysis; SVO, small‐vessel occlusion; TOAST, Trial of ORG 10172 in Acute Stroke Treatment.

12.85% (1657) participants had poor functional status and 2.87% (380) deaths occurred during 1 year. A previous survey from the Bigdata Observatory Platform for Stroke of China (BOSC) revealed that the rate of 1‐year death and disability was 6.0% (5.7%−6.3%) and 14.2% (13.8%−14.7%) respectively after first‐ever stroke in Chinese population. Although compared to the BOSC our study was with higher mortality, study cohorts and inclusion criteria like whether the first‐stroke or not and the occurrence‐to‐admission time could explain the reason (Tu et al., 2021). Compared with the first quartile, the multivariable adjusted ORs and 95% CIs for the risk of poor functional outcome gradually decreased in the higher quartile groups (second quartile (Q2), adjusted OR [aOR], 0.70 [95% CI, 0.58–0.83]; third quartile (Q3), aOR, 0.53 [95% CI, 0.42–0.65]; fourth quartile (Q4), aOR, 0.43 [95% CI, 0.33–0.56], *p* for trend < .001). Similarly, the significant association was also found when examining mRS as an ordinary variable (Q1 vs. Q4, 0.80 [95% CI 0.72–0.89] vs. 0.60 [95% CI 0.52–0.70]). A 100 kcal/day increment of predicted RMR was inversely associated with poor functional outcome (aOR, 0.78 [95% CI 0.74–0.83]) (Table 2). We also examined the relationship between predicted RMR and poor functional outcome at 3‐month, the similar results were obtained (Supplemental Table S3)

**TABLE 2:** Association between predicted RMR, poor functional outcome, ordinary mRS, and all‐cause mortality

		Unadjusted	*p* Value	Sex adjusted	*p* Value	Multivariable adjusted^a^	*p* Value	*p* for trend
		OR/HR (95% CI)		OR/HR (95% CI)		OR/HR (95% CI)		
mRS ≥3	Q 1	Ref.^b^ (1)		Ref. (1)		Ref. (1)		<.001
	Q 2	0.74 (0.64–0.84)	<.0001	0.70 (0.61–0.81)	<.0001	0.70 (0.58–0.83)	<.0001	
	Q 3	0.59 (0.51–0.68)	<.0001	0.54 (0.46–0.64)	<.0001	0.53 (0.42–0.65)	<.0001	
	Q 4	0.49 (0.42–0.56)	<.0001	0.44 (0.37–0.52)	<.0001	0.43 (0.33–0.56)	<.0001	
Ordinary mRS	Q 1	Ref. (1)		Ref. (1)		Ref. (1)		<.001
	Q 2	0.82 (0.75–0.89)	<.0001	0.78 (0.71–0.85)	<.0001	0.80 (0.72–0.89)	<.0001	
	Q 3	0.72 (0.66–0.79)	<.0001	0.66 (0.59–0.73)	<.0001	0.69 (0.61–0.78)	<.0001	
	Q 4	0.63 (0.57–0.69)	<.0001	0.56 (0.50–0.63)	<.0001	0.60 (0.52–0.70)	<.0001	
Per 100 kcal/day increment of RMR for mRS ≥3		0.85 (0.83–0.88)	<.0001	0.83 (0.80–0.86)	<.0001	0.78 (0.74–0.83)	<.0001	
All‐cause mortality	Q 1	Ref. (1)		Ref. (1)		Ref. (1)		<.001
	Q 2	0.68 (0.53–0.89)	.0042	0.60 (0.46–0.79)	.0003	0.69 (0.51–0.94)	.02	
	Q 3	0.61 (0.47–0.80)	.0003	0.48 (0.35–0.66)	<.0001	0.58 (0.40–0.85)	.0055	
	Q 4	0.42 (0.31–0.57)	<.0001	0.32 (0.23–0.50)	<.0001	0.44 (0.28–0.71)	.0008	
Per 100 kcal/day increment of RMR for all‐cause mortality		0.83 (0.79–0.88)	<.0001	0.78 (0.73–0.84)	<.0001	0.81 (0.73–0.89)	<.0001	

^a^ Adjusted by sex, BMI, current smoking, hypertension, dyslipidemia, diabetes mellitus, prior stroke, prior atrial fibrillation, prior coronary heart disease, NIHSS, and mRS at admission, TOAST subtype, recombinant tissue plasminogen activator intravenous thrombolysis treatment, antithrombotic drugs, and lipid‐lowering drug.

^b^ Ref. indicates reference.

Abbreviations: HR, hazard ratio; mRS, modified Rankin Scale; OR, odds ratio; RMR, resting metabolic rate.

**FIGURE 1:**
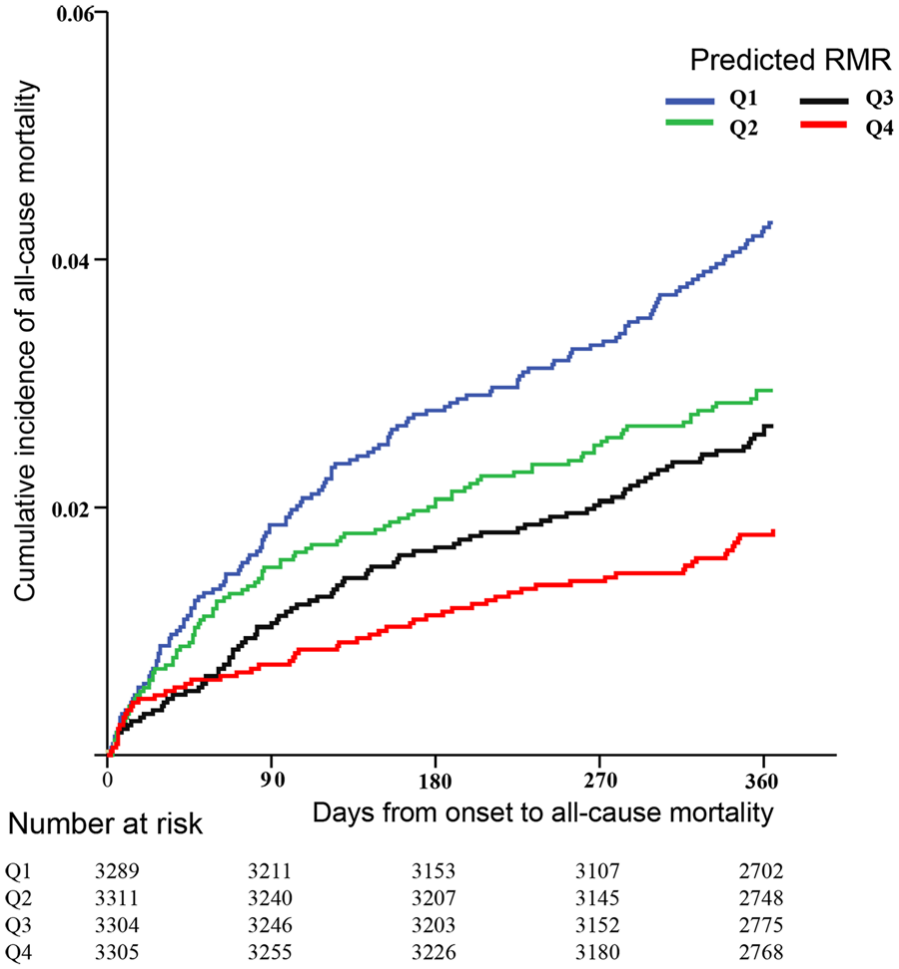
Kaplan–Meier curves for incidence of all‐cause mortality by quartiles of predicted RMR.

The inverse relationship was also observed between the predicted RMR and all‐cause mortality (Q2, adjusted HR [aHR], 0.69 [95% CI, 0.51–0.94]; Q3, aHR, 0.58 [95% CI, 0.40–0.85]; Q4, aHR, 0.44 [95% CI, 0.28–0.71]). An all‐cause mortality risk reduction of 19% was associated with each 100 kcal/day increment in predicted RMR (aHR, 0.81 [95% CI 0.73–0.89]) (Table 2). Similarly, at 3 month the predicted RMR still showed the inverse association with all‐cause mortality, although which turned nonsignificant after adjusting the multiple confounders due to certain potential factors (Supplemental Table S3). The cumulative incidence of all‐cause mortality was significantly lower in participants with the highest predicted RMR (Q1 versus Q4, 4.2% versus 1.8%, *p* < .0001) (Figure 1).

**FIGURE 2:**
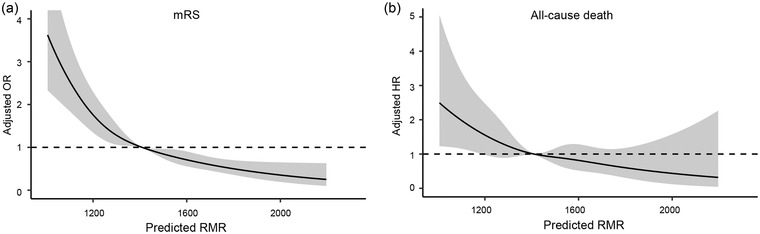
Restricted cubic spline for poor functional outcome (A) and all‐cause mortality (B) according to levels of predicted RMR. Solid dark line is a multivariable adjusted OR/HR, with a gray area indicating 95% CI derived from restricted cubic spline regression. A dashed dark line represents no association.

The restricted cubic spline model indicated a similar associated pattern to the predicted RMR as a categorical variable both in the relationship with poor functional status and all‐cause mortality (Figure 2). No significant heterogeneity was observed across specified subgroups of age, sex, and BMI on the primary endpoints (all *p* for interaction >.05; Supplemental Table S4). The sensitivity analysis performed in 12,914 participants of mRS of 0 to 3 did not change the above reverse associations (Supplemental Table S5).

## 5 DISCUSSION

This study provided that after controlling for related confounders, predicted RMR was significantly inversely associated with poor functional status and all‐cause mortality. Furthermore, the results were consistent even after stratification for age, sex, and BMI. The results indicated that predicted RMR seemed to be an independent protective predictor for prognosis of ischemic stroke as a proxy of metabolic profile.

Given that the RMR measured in current study was only based on the age, weight, and height variable by sex, so in theory, the male subjects with bigger body size (greater weight and higher) and younger age were more likely to obtain good outcome after ischemic stroke. Although it seemed to appear that outcome status was decided on the pure sum of the variables in equation, we should note that each of the variables has different weighted factors which indicted that different RMR would be obtained by changes in same unit of every variable. Owning to measurements on RMR obtained by Harris–Benedict equation (closely associated with indirect calorimetry in nonobesity, healthy participants and most subjects in this study satisfied these conditions as presented in Table 1) instead of indirect calorimetry, with consideration of its significance in clinical practice, further testified studies on stroke and RMR with an indirect calorimetry method were expected.

Using RMR as a potential biomarker of body metabolic health has been supported by several previous studies. However, the conclusion drawn on the dangerous indicator of higher RMR for diseases or death appeared to predominate over its protective side (Drabsch et al., 2018; Hand & Blair, 2014; Jumpertz et al., 2011; Kang et al., 2021; Kliemann et al., 2020; Ruggiero et al., 2008; Schrack et al., 2014). An over 40‐year follow‐up of 1227 healthy participants conducted in the Baltimore Longitudinal Study of Aging (BLSA), mostly men, indicating those with high basal energy rate was associated with shorter longevity. (Ruggiero et al., 2008) That was supported by an over 2‐year calorie restriction trial on 53 nonobese adults showing that decreased energy expenditure improved the rate of living. (Redman et al., 2018) Higher incidences of urolithiasis recurrence and diabetes were observed in the individuals with higher RMR (Kang et al., 2021). Nonetheless, diabetes and metabolic syndrome were also found more likely to occur in the population with low RMR (Buscemi et al., 2007; Georgopoulos et al., 2009; Maciak et al., 2020; Olive et al., 2008). Our results seemed to disagree with the tendency of the dangerous implication of RMR for healthy state. The target population among studies, like whether they were healthy, sex‐specific dominant, obese, with ischemic stroke or not, had different demographic characteristics. In addition, difference in study design and RMR evaluating methods among studies could partly account for the discrepancy of conclusions. Our interests in terms of secondary prevention instead of primary prevention could cause the appearance of protective effect that may be similar to the relationship between obesity and ischemic stroke (Vemmos et al., 2011). The controversial relationship between RMR and oxidative stress that is considered to damage health might be one of the inconsistent causes (Frisard & Ravussin, 2006). Therefore, to confirm our findings, further studies evaluating populations affected by stroke or other diseases are warranted.

A consistent tendency between the level of predicted RMR and BMI (*p* < .0001) in our study may result from the common crucial variable of weight (Table 1). In the subgroup analysis of overweight/obesity, our results found predicted RMR could act as an independent protective factor for the poor functional outcome (*p* for interaction, .24) and all‐cause mortality (*p* for interaction, .84) (Supplemental Table S3). This indicated that predicted RMR could detect extra information beyond BMI, while BMI is positively associated with improved outcome after ischemic stroke (Vemmos et al., 2011).

Although scarce studies directly examined the protective predictive value of RMR on unfavorable ischemic stroke prognosis, several mechanisms could account for the inverse association. The positive relationship between RMR and cardiorespiratory fitness has been found, and the latter is treated as a target to reduce cardiovascular diseases and mortality. Higher RMR could represent better cardiorespiratory fitness and promotes functional recovery (Ebaditabar et al., 2021; Shook et al., 2014). On the other hand, higher RMR represents greater composition of skeletal muscle, the loss of which significantly influences the physical activity (Li et al., 2020; Oh et al., 2019; Soysal et al., 2019; Visser et al., 2005). Additionally, a series of spontaneous recovery processes after ischemic stroke, such as the activity of neuroglial cells and the changes of repair‐related molecular, need support of the strong reverse capacity that may be along with great RMR (Bélanger et al., 2011; Cramer, 2008; Zampino et al., 2020).

The study first applied a large‐scale cohort with ischemic stroke to examine the association between energy metabolism and ischemic stroke prognosis. However, some limitations should be considered. First, the RMR was predicted by the equation instead of a gold standard with indirect calorimetry owing to the expensive prices and complex operation. A decrease in accuracy of the Harris–Benedict equation may occur when used in individuals with low or high BMI and older age (Jésus et al., 2015). Given that, we excluded the patient whose age or BMI was beyond the range from 30 to 80 and from 16 to 40, respectively. Second, the predicted RMR was measured once at baseline regardless of its potential change over time. Third, the information on the self‐reported thyroid disease lacked in our analysis. Concerning that the prevalence of overt hyperthyroidism and hypothyroidism in a nationwide survey with an enrollment of 78, 470 Chinese adults was 0.78% (95% CI, 0.69% to 0.87%) and 1.02% (95% CI, 0.88% to 1.18%) respectively, our results might not be influenced substantially (Li et al., 2020). Fourth, there were a lot of potential confounders like nutritional status, body composition and etc. which we did not assess due to lack of necessary related data, that could interfere the outcomes. Finally, relatively short follow‐up period and small cumulative number of deaths (380, 2.87%) led to no death in female patient with highest predicted RMR, which could cause a lower statistical power in assessing the association between predicted RMR and outcome.

In summary, higher predicted RMR could be an independent protective indicator for the risk of poor functional outcome and all‐cause mortality of ischemic stroke as a proxy of metabolic profile. Nevertheless, it is not yet known whether the RMR is merely a proxy or plays a causal role in the relationship with poststroke outcome. Figuring it out will facilitate to improve outcome and decrease death and disability by changing the RMR. We here proposed the following potential points regarding the further study on RMR: use of the more rigorous methods to verify the association like golden standard, study of the molecular and cellular mechanisms that influence RMR so as to seek to transform it into pharmaceutical and behavioral intervention.

## AUTHOR CONTRIBUTIONS

XYL and ACC drafted the manuscript and interpreted the data. YSP and MXW contributed to revised the manuscript and statistical problems. XM revised the manuscript. YJW interpreted data and revised the manuscript. All authors read and approved the final manuscript to be published.

## CONFLICT OF INTEREST STATEMENT

The author declares that there is no conflict of interest that could be perceived as prejudicing the impartiality of the research reported.

### PEER REVIEW

The peer review history for this article is available at https://publons.com/publon/10.1002/brb3.2911

## Supporting information

Table S1. Predicted resting metabolic rate equations.
Table S2. Pearson's correlation coefficients of Harris–Benedict equation, Mifflin St Jeor, WHO/FAO/UNU, and Oxford equations.
Table S3. Association between predicted RMR, poor functional outcome, ordinary mRS, and all‐cause mortality at 3 months.
Table S4. Subgroup analysis of predicted RMR, poor functional outcome (mRS ≥3) (a), and all‐cause mortality (b).
Table S5. Sensitivity analysis of predicted RMR, poor functional outcome, ordinary mRS, and all‐cause mortality.

## Data Availability

All data are available to researchers on request for purposes of reproducing the results or replicating the procedure by directly contacting the corresponding author.

## References

[brb32911-bib-0001] Bélanger, M. , Allaman, I. , & Magistretti, P. J. (2011). Brain energy metabolism: Focus on astrocyte‐neuron metabolic cooperation. Cell Metabolism, 14(6), 724–738. 10.1016/j.cmet.2011.08.016 22152301

[brb32911-bib-0002] Bendavid, I. , Lobo, D. N. , Barazzoni, R. , Cederholm, T. , Coëffier, M. , De Van Der Schueren, M. , Fontaine, E. , Hiesmayr, M. , Laviano, A. , Pichard, C. , & Singer, P. (2021). The centenary of the Harris–Benedict equations: How to assess energy requirements best? Recommendations from the ESPEN expert group. Clinical Nutrition, 40(3), 690–701. 10.1016/j.clnu.2020.11.012 33279311

[brb32911-bib-0003] Buscemi, S. , Verga, S. , Caimi, G. , & Cerasola, G. (2007). A low resting metabolic rate is associated with metabolic syndrome. Clinical Nutrition, 26(6), 806–809. 10.1016/j.clnu.2007.08.010 17936441

[brb32911-bib-0004] Cramer, S. C. (2008). Repairing the human brain after stroke: I. Mechanisms of spontaneous recovery. Annals of Neurology, 63(3), 272–287. 10.1002/ana.21393 18383072

[brb32911-bib-0005] Drabsch, T. , Holzapfel, C. , Stecher, L. , Petzold, J. , Skurk, T. , & Hauner, H. (2018). Associations between C‐reactive protein, insulin sensitivity, and resting metabolic rate in adults: A mediator analysis. Frontiers in Endocrinology (Lausanne), 9, 556. 10.3389/fendo.2018.00556 PMC6158372 30294302

[brb32911-bib-0006] Ebaditabar, M. , Imani, H. , Babaei, N. , Davarzani, S. , & Shab‐Bidar, S. (2021). Maximal oxygen consumption is positively associated with resting metabolic rate and better body composition profile. Obesity Medicine, 21, 100309. 10.1016/j.obmed.2020.100309

[brb32911-bib-0007] Energy and Protein Requirements . (1985). Report of a joint FAO/WHO/UNU expert consultation. World Health Organization Technical Report Series, 724, 1–206. 3937340

[brb32911-bib-0008] Fabbri, E. , An, Y. , Schrack, J. A. , Gonzalez‐Freire, M. , Zoli, M. , Simonsick, E. M. , Guralnik, J. M. , Boyd, C. M. , Studenski, S. A. , & Ferrucci, L. (2015). Energy metabolism and the burden of multimorbidity in older adults: Results from the Baltimore Longitudinal Study of Aging. Journals of Gerontology. Series A, Biological Sciences and Medical Sciences, 70(11), 1297–1303. 10.1093/gerona/glu209 25409892 PMC4612383

[brb32911-bib-0009] Frisard, M. , & Ravussin, E. (2006). Energy metabolism and oxidative stress: Impact on the metabolic syndrome and the aging process. Endocrine, 29(1), 27–32. 10.1385/endo:29:1:27 16622290

[brb32911-bib-0010] Georgopoulos, N. A. , Saltamavros, A. D. , Vervita, V. , Karkoulias, K. , Adonakis, G. , Decavalas, G. , Kourounis, G. , Markou, K. B. , & Kyriazopoulou, V. (2009). Basal metabolic rate is decreased in women with polycystic ovary syndrome and biochemical hyperandrogenemia and is associated with insulin resistance. Fertility and Sterility, 92(1), 250–255. 10.1016/j.fertnstert.2008.04.067 18678372

[brb32911-bib-0011] Hand, G. A. , & Blair, S. N. (2014). Energy flux and its role in obesity and metabolic disease. European Journal of Endocrinology, 10(2), 131–135. 10.17925/ee.2014.10.02.131 PMC5983082 29872477

[brb32911-bib-0012] Harris, J. A. , & Benedict, F. G. (1918). A biometric study of human basal metabolism. Proceedings of the National Academy of Sciences of the United States of America, 4(12), 370–373. 10.1073/pnas.4.12.370 16576330 PMC1091498

[brb32911-bib-0013] Henry, C. (2005). Basal metabolic rate studies in humans: Measurement and development of new equations. Public Health Nutrition, 8(7a), 1133–1152. 10.1079/phn2005801 16277825

[brb32911-bib-0014] Jésus, P. , Achamrah, N. , Grigioni, S. , Charles, J. , Rimbert, A. , Folope, V. , Petit, A. , Déchelotte, P. , & Coëffier, M. (2015). Validity of predictive equations for resting energy expenditure according to the body mass index in a population of 1726 patients followed in a nutrition unit. Clinical Nutrition, 34(3), 529–535. 10.1016/j.clnu.2014.06.009 25016971

[brb32911-bib-0015] Jumpertz, R. , Hanson, R. L. , Sievers, M. L. , Bennett, P. H. , Nelson, R. G. , & Krakoff, J. (2011). Higher energy expenditure in humans predicts natural mortality. Journal of Clinical Endocrinology and Metabolism, 96(6), E972–E976. 10.1210/jc.2010-2944 21450984 PMC3100751

[brb32911-bib-0016] Kang, H. W. , Seo, S. P. , Lee, H. Y. , Kim, K. , Ha, Y. ‐S. , Kim, W. T. , Kim, Y. ‐J. , Yun, S. ‐J. , Kim, W. ‐J. , & Lee, S. ‐C. (2021). A high basal metabolic rate is an independent predictor of stone recurrence in obese patients. Investigative and Clinical Urology, 62(2), 195–200. 10.4111/icu.20200438 33660447 PMC7940852

[brb32911-bib-0017] Kliemann, N. , Murphy, N. , Viallon, V. , Freisling, H. , Tsilidis, K. K. , Rinaldi, S. , Mancini, F. R. , Fagherazzi, G. , Boutron‐Ruault, M.‐C. , Boeing, H. , Schulze, M. B. , Masala, G. , Krogh, V. , Sacerdote, C. , Magistris, M. S. , Bueno‐De‐Mesquita, B. , Weiderpass, E. , Kühn, T. , Kaaks, R. , … Gunter, M. J. (2020). Predicted basal metabolic rate and cancer risk in the European Prospective Investigation into cancer and nutrition. International Journal of Cancer, 147(3), 648–661. 10.1002/ijc.32753 31652358

[brb32911-bib-0018] Lau, L.‐H. , Lew, J. , Borschmann, K. , Thijs, V. , & Ekinci, E. I. (2019). Prevalence of diabetes and its effects on stroke outcomes: A meta‐analysis and literature review. Journal of Diabetes Investigation, 10(3), 780–792. 10.1111/jdi.12932 30220102 PMC6497593

[brb32911-bib-0019] Li, W. , Yue, T. , & Liu, Y. (2020). New understanding of the pathogenesis and treatment of stroke‐related sarcopenia. Biomedicine & Pharmacotherapy, 131, 110721. 10.1016/j.biopha.2020.110721 32920517

[brb32911-bib-0020] Li, Y. , Teng, D. i. , Ba, J. , Chen, B. , Du, J. , He, L. , Lai, X. , Teng, X. , Shi, X. , Li, Y. , Chi, H. , Liao, E. , Liu, C. , Liu, L. , Qin, G. , Qin, Y. , Quan, H. , Shi, B. , Sun, H. , … Teng, W. (2020). Efficacy and safety of long‐term universal salt iodization on thyroid disorders: Epidemiological evidence from 31 provinces of Mainland China. Thyroid: Official Journal of the American Thyroid Association, 30(4), 568–579. 10.1089/thy.2019.0067 32075540

[brb32911-bib-0021] Maciak, S. , Sawicka, D. , Sadowska, A. , Sadowska, A. , Prokopiuk, S. , Buczyńska, S. , Bartoszewicz, M. , Niklińska, G. , Konarzewski, M. , & Car, H. (2020). Low basal metabolic rate as a risk factor for development of insulin resistance and type 2 diabetes. BMJ Open Diabetes Research & Care, 8(1), 10.1136/bmjdrc-2020-001381 PMC7373309 32690630

[brb32911-bib-0022] Mifflin, M. D. , St Jeor, S. T. , Hill, L. A. , Scott, B. J. , Daugherty, S. A. , & Koh, Y. O. (1990). A new predictive equation for resting energy expenditure in healthy individuals. American Journal of Clinical Nutrition, 51(2), 241–247. 10.1093/ajcn/51.2.241 2305711

[brb32911-bib-0023] Oh, S.‐K. , Son, D. a.‐H. , Kwon, Y. u.‐J. , Lee, H. S. , & Lee, J. i.‐W. (2019). Association between basal metabolic rate and handgrip strength in older Koreans. International Journal of Environmental Research and Public Health, 16(22), 4377. 10.3390/ijerph16224377 31717481 PMC6888346

[brb32911-bib-0024] Olive, J. L. , Ballard, K. D. , Miller, J. J. , & Milliner, B. A. (2008). Metabolic rate and vascular function are reduced in women with a family history of type 2 diabetes mellitus. Metabolism, 57(6), 831–837. 10.1016/j.metabol.2008.01.028 18502267

[brb32911-bib-0025] Redman, L. M. , Smith, S. R. , Burton, J. H. , Martin, C. K. , Il'yasova, D. , & Ravussin, E. (2018). Metabolic slowing and reduced oxidative damage with sustained caloric restriction support the rate of living and oxidative damage theories of aging. Cell Metabolism, 27(4), 805–815.e4. 10.1016/j.cmet.2018.02.019 29576535 PMC5886711

[brb32911-bib-0026] Rodríguez‐Castro, E. , Rodríguez‐Yáñez, M. , Arias‐Rivas, S. , Santamaría‐Cadavid, M. , López‐Dequidt, I. , Hervella, P. , López, M. , Campos, F. , Sobrino, T. , & Castillo, J. (2019). Obesity paradox in ischemic stroke: Clinical and molecular insights. Translational Stroke Research, 10(6), 639–649. 10.1007/s12975-019-00695-x 30980283

[brb32911-bib-0027] Ruggiero, C. , Metter, E. J. , Melenovsky, V. , Cherubini, A. , Najjar, S. S. , Ble, A. , Senin, U. , Longo, D. L. , & Ferrucci, L. (2008). High basal metabolic rate is a risk factor for mortality: The Baltimore Longitudinal Study of Aging. Journals of Gerontology. Series A, Biological Sciences and Medical Sciences, 63(7), 698–706. 10.1093/gerona/63.7.698 18693224 PMC4984846

[brb32911-bib-0028] Schrack, J. A. , Knuth, N. D. , Simonsick, E. M. , & Ferrucci, L. (2014). IDEAL” aging is associated with lower resting metabolic rate: The Baltimore Longitudinal Study of Aging. Journal of the American Geriatrics Society, 62(4), 667–672. 10.1111/jgs.12740 24635835 PMC3989425

[brb32911-bib-0029] Shook, R. P. , Hand, G. A. , Paluch, A. E. , Wang, X. , Moran, R. , Hébert, J. R. , Lavie, C. J. , & Blair, S. N. (2014). Moderate cardiorespiratory fitness is positively associated with resting metabolic rate in young adults. Mayo Clinic Proceedings, 89(6), 763–771. 10.1016/j.mayocp.2013.12.017 24809761

[brb32911-bib-0030] Soysal, P. , Ates Bulut, E. , Yavuz, I. , & Isik, A. T. (2019). Decreased basal metabolic rate can be an objective marker for sarcopenia and frailty in older males. Journal of the American Medical Directors Association, 20(1), 58–63. 10.1016/j.jamda.2018.07.001 30122323

[brb32911-bib-0031] Tu, W. ‐. J. , Chao, B. ‐. H. , Ma, L. , Yan, F. , Cao, L. , Qiu, H. , Ji, X. ‐. M. , & Wang, L.‐D. e (2021). Case‐fatality, disability and recurrence rates after first‐ever stroke: A study from bigdata observatory platform for stroke of China. Brain Research Bulletin, 175, 130–135. 10.1016/j.brainresbull.2021.07.020 34329730

[brb32911-bib-0032] Vemmos, K. , Ntaios, G. , Spengos, K. , Savvari, P. , Vemmou, A. , Pappa, T. , Manios, E. , Georgiopoulos, G. , & Alevizaki, M. (2011). Association between obesity and mortality after acute first‐ever stroke: The obesity‐stroke paradox. Stroke; A Journal of Cerebral Circulation, 42(1), 30–36. 10.1161/strokeaha.110.593434 21127299

[brb32911-bib-0033] Visser, M. , Goodpaster, B. H. , Kritchevsky, S. B. , Newman, A. B. , Nevitt, M. , Rubin, S. M. , Simonsick, E. M. , & Harris, T. B. (2005). Muscle mass, muscle strength, and muscle fat infiltration as predictors of incident mobility limitations in well‐functioning older persons. Journals of Gerontology. Series A, Biological Sciences and Medical Sciences, 60(3), 324–333. 10.1093/gerona/60.3.324 15860469

[brb32911-bib-0034] Wang, Y. , Jing, J. , Meng, X. , Pan, Y. , Wang, Y. , Zhao, X. , Lin, J. , Li, W. , Jiang, Y. , Li, Z. , Zhang, X. , Yang, X. , Ji, R. , Wang, C. , Wang, Z. , Han, X. , Wu, S. , Jia, Z. , Chen, Y. , & Li, H. (2019). The Third China National Stroke Registry (CNSR‐III) for patients with acute ischaemic stroke or transient ischaemic attack: Design, rationale and baseline patient characteristics. Stroke and Vascular Neurology, 4(3), 158–164. 10.1136/svn-2019-000242 31709123 PMC6812638

[brb32911-bib-0035] Wang, Y. J. , Li, Z. X. , Gu, H. Q. , Zhai, Y. , Jiang, Y. , Zhao, X.‐Q. , Wang, Y.‐L. , Yang, X. , Wang, C.‐J. , Meng, X. , Li, H. , Liu, L.‐P. , Jing, J. , Wu, J. , Xu, A.‐D. , Dong, Q. , Wang, D. , Zhao, J.‐Z. , & China Stroke Statistics 2019 Writing Committee . (2020). China stroke statistics 2019: A report from the National Center for Healthcare Quality Management in Neurological Diseases, China National Clinical Research center for neurological diseases, the Chinese Stroke Association, National Center for Chronic and Non‐Communicable Disease Control and Prevention, Chinese Center for Disease Control and Prevention and Institute for Global Neuroscience and Stroke Collaborations. Stroke and Vascular Neurology, 5(3), 211–239. 10.1136/svn-2020-000457 32826385 PMC7548521

[brb32911-bib-0036] Yang, S. , Boudier‐Revéret, M. , Kwon, S. , Lee, M. Y. , & Chang, M. C. (2021). Effect of diabetes on post‐stroke recovery: A systematic narrative review. Frontiers in Neurology, 12, 747878. 10.3389/fneur.2021.747878 34970205 PMC8712454

[brb32911-bib-0037] Zampino, M. , Alghatrif, M. , Kuo, P. ‐L. , Simonsick, E. M. , & Ferrucci, L. (2020). Longitudinal changes in resting metabolic rates with aging are accelerated by diseases. Nutrients, 12(10), 3061. 10.3390/nu12103061 33036360 PMC7600750

