# Supplementary material for: Predicted resting metabolic rate and prognosis in patients with ischemic stroke
Source: Brain Behav. 2023 Feb 7;13(3):e2911. doi: 10.1002/brb3.2911 (PMC10013948; doi:10.1002/brb3.2911)
Supplement: Supplementary file 1 — Table S1. Predicted resting metabolic rate equations. Table S2. Pearson's correlation coefficients of Harris–Benedict equation, Mifflin St Jeor, WHO/FAO/UNU, and Oxford equations. Table S3. Association between predicted RMR, poor functional outcome, ordinary mRS, and all‐cause mortality at 3 months. Table S4. Subgroup analysis of predicted RMR, poor functional outcome (mRS ≥3) (a), and all‐cause mortality (b). Table S5. Sensitivity analysis of predicted RMR, poor functional outcome, ordinary mRS, and all‐cause mortality. [file BRB3-13-e2911-s001.docx]

Supplementary Table 1. Predicted resting metabolic rate equations

|  | Men | Women |
| --- | --- | --- |
| Harris-Benedict | 66+ (13.7×weight in kg) + (5 × height in cm) - (6.8× age in year) | 655+ (9.6× weight) + (1.8× height in cm) - (4.7× age in year) |
| WHO/FAO/UNU |  |  |
| 18-30 years | 15.4× weight in kg– 27× height in cm+ 717 | 13.3× weight in kg+ 334× height in cm+ 35 |
| 31-60 years | 11.3× weight in kg+ 16× height in cm+ 901 | 8.7× weight in kg-25× height in cm+ 865 |
| >60 years | 8.8× weight in kg+ 1128× height in cm- 1071 | 9.2× weight in kg+ 637× height in cm- 302 |
| Oxford |  |  |
| 18-30 years | 14.4× weight in kg+ 313× height in cm/100+ 113 | 10.4× weight in kg+ 615× height in cm/100- 282 |
| 31-60 years | 11.4× weight in kg+ 541× height in cm/100- 137 | 8.18× weight in kg+ 502× height in cm/100- 11.6 |
| >60 years | 11.4× weight in kg+ 541× height in cm/100- 256 | 8.52× weight in kg+ 421× height in cm/100+ 10.7 |
| Mifflin St Jeor | (10× weight in kg) + (6.25× height in cm) - (5× age in year) + 5 | (10× weight in kg) + (6.25× height in cm) - (5× age in year) - 161 |

Supplementary Table 2. Pearson correlation coefficients of Harris-Benedict equation, Mifflin St Jeor, WHO/FAO/UNU, and Oxford equations

| P value R² | Harris-Benedict | Mifflin St Jeor | WHO/FAO/UNU | Oxford |
| --- | --- | --- | --- | --- |
| Harris-Benedict |  | 0.96 | 0.92 | 0.96 |
| Mifflin St Jeor | P<0.0001 |  | 0.97 | 0.98 |
| WHO/FAO/UNU | P<0.0001 | P<0.0001 |  | 0.99 |
| Oxford | P<0.0001 | P<0.0001 | P<0.0001 |  |

|  |  | Unadjusted | P value | Sex adjusted | P value | Multivariable adjusted ¶ | P value | P for trend |
| --- | --- | --- | --- | --- | --- | --- | --- | --- |
|  |  | OR/HR (95%CI) |  | OR/HR (95%CI) |  | OR/HR (95%CI) |  |  |
| mRS ≥3 | Q 1 | Ref. § (1) |  | Ref. (1) |  | Ref. (1) |  | <0.0001 |
|  | Q 2 | 0.71(0.62-0.81) | <0.0001 | 0.71(0.62-0.82) | <0.0001 | 0.72(0.60-0.85) | 0.0002 |  |
|  | Q 3 | 0.59(0.52-0.68) | <0.0001 | 0.59(0.50-0.70) | <0.0001 | 0.62(0.50-0.77) | <0.0001 |  |
|  | Q 4 | 0.56(0.48-0.65) | <0.0001 | 0.56(0.47-0.66) | <0.0001 | 0.59(0.46-0.77) | <0.0001 |  |
| Ordinary mRS | Q 1 | Ref. (1) |  | Ref. (1) |  | Ref. (1) |  | <0.0001 |
|  | Q 2 | 0.82(0.75-0.90) | <0.0001 | 0.81(0.73-0.89) | <0.0001 | 0.72(0.60-0.85) | 0.0002 |  |
|  | Q 3 | 0.74(0.68-0.81) | <0.0001 | 0.71(0.64-0.79) | <0.0001 | 0.62(0.50-0.77) | <0.0001 |  |
|  | Q 4 | 0.67(0.61-0.72) | <0.0001 | 0.64(0.57-0.72) | <0.0001 | 0.59(0.46-0.77) | <0.0001 |  |
| per 100 Kcal/d increment of RMR for mRS ≥3 | | 0.88(0.86-0.90) | <0.0001 | 0.88(0.85-0.91) | <0.0001 | 0.86(0.82-0.91) | <0.0001 |  |
| All-cause mortality | Q 1 | Ref. (1) |  | Ref. (1) |  | Ref. (1) |  | <0.0001 |
|  | Q 2 | 0.82(0.56-1.18) | 0.28 | 0.73(0.49-1.09) | 0.12 | 0.96(0.61-1.52) | 0.86 |  |
|  | Q 3 | 0.57(0.38-0.86) | 0.0081 | 0.47(0.29-0.76) | 0.0019 | 0.72(0.40-1.28) | 0.26 |  |
|  | Q 4 | 0.39(0.24-0.63) | <0.0001 | 0.32(0.19-0.54) | <0.0001 | 0.61(0.30-1.25) | 0.18 |  |
| per 100 Kcal/d increment of RMR for all-cause mortality | | 0.81(0.74-0.88) | <0.0001 | 0.76(0.69-0.84) | <0.0001 | 0.83(0.71-0.97) | 0.02 |  |

Supplementary Table 3. Association between predicted RMR, poor functional outcome, ordinary mRS, and all-cause mortality at 3-month

§Ref. indicates reference

¶ Adjusted by sex, BMI, current smoking, hypertension, dyslipidemia, diabetes mellitus, prior stroke, prior atrial fibrillation, prior coronary heart disease, NIHSS, and mRS at admission, TOAST subtype, recombinant tissue plasminogen activator intravenous thrombolysis treatment, antithrombotic drugs, and lipid-lowering drug

Abbreviations: RMR; resting metabolic rate; mRS, modified Rankin Scale; OR, odds ratio; HR, hazard ratio;

Supplementary Table 4. Subgroup analysis of predicted RMR, poor functional outcome (mRS ≥3) (a), and all-cause mortality (b)

1. Poor functional outcome

|  | Predicted Resting metabolic rate (RMR) (Kcal/d) | | | |  |
| --- | --- | --- | --- | --- | --- |
|  | Q1 | Q2 | Q3 | Q4 |  |
| Subgroup | Events (n/%) | Events (n/%) | Events (n/%) | Events (n/%) | P for interaction |
| Age |  | | | | 0.14 |
| ≤60 | 61(10.7) | 116(9.5) | 130(8.6) | 169(7.0) |  |
| OR (95%CI) | Ref.§ (1) | 0.87(0.58-1.30) | 0.73(0.45-1.19) | 0.59(0.33-1.03) |  |
| >60 | 500(18.9) | 320(16.0) | 229(13.5) | 132(16.2) |  |
| OR (95%CI) | Ref. (1) | 0.82(0.66-1.02) | 0.65(0.50-0.86) | 0.78(0.55-1.12) |  |
| BMI |  | | | | 0.24 |
| <25 | 460(17.2) | 281(14.0) | 187(9.7) | 56(8.0) |  |
| OR (95%CI) | Ref. (1) | 0.77(0.63-0.95) | 0.52(0.41-0.66) | 0.45(0.32-0.64) |  |
| ≥25 | 101(19.1) | 155(12.7) | 172(13.3) | 245(9.7) |  |
| OR (95%CI) | Ref. (1) | 0.62(0.45-0.84) | 0.56(0.38-0.82) | 0.39(0.26-0.58) |  |
| Sex |  | | | | 0.36 |
| man | 144(17.4) | 290(14.9) | 332(11.2) | 299(9.3) |  |
| OR (95%CI) | Ref. (1) | 0.80(0.62-1.02) | 0.56(0.43-0.73) | 0.46(0.33-0.62) |  |
| woman | 417(17.5) | 146(11.4) | 27(10.3) | 2(6.9) |  |
| OR (95%CI) | Ref. (1) | 0.62(0.47-0.80) | 0.56(0.33-0.94) | 0.29(0.06-1.39) |  |

§Ref. indicates reference

1. All-cause mortality

|  | Predicted Resting metabolic rate (RMR)(Kcal/d) | | | |  |
| --- | --- | --- | --- | --- | --- |
|  | Q1 | Q2 | Q3 | Q4 |  |
| Subgroup | Events (n/%) | Events (n/%) | Events (n/%) | Events (n/%) | P for interaction |
| Age |  | | | | 0.61 |
| ≤60 | 11(1.9) | 15(1.2) | 25(1.6) | 33(1.3) |  |
| HR (95%CI) | Ref. (1) | 0.70(0.28-1.75) | 0.80(0.28-2.26) | 0.81(0.25-2.69) |  |
| >60 | 128(4.7) | 81(3.9) | 61(3.5) | 26(3.1) |  |
| HR (95%CI) | Ref. (1) | 0.90(0.63-1.29) | 0.80(0.51-1.26) | 0.70(0.38-1.29) |  |
| BMI |  | | | | 0.84 |
| <25 | 120(4.4) | 63(3.1) | 49(2.5) | 14(1.9) |  |
| HR (95%CI) | Ref. | 0.64(0.45-0.92) | 0.54(0.36-0.81) | 0.47(0.26-0.86) |  |
| ≥25 | 19(3.5) | 33(2.7) | 37(2.8) | 45(1.7) |  |
| HR (95%CI) | Ref. | 0.67(0.38-1.21) | 0.44(0.22-0.91) | 0.27(0.13-0.57) |  |
| Sex |  | | | | 0.54 |
| man | 40(4.7) | 73(3.6) | 80(2.6) | 59(1.8) |  |
| HR (95%CI) | Ref. | 0.77(0.51-1.16) | 0.58(0.37-0.91) | 0.43(0.24-0.75) |  |
| woman | 99(4.1) | 23(1.8) | 6(2.3) | 0(0) |  |
| HR (95%CI) | Ref. | 0.59(0.35-0.99) | 0.80(0.29-2.18) | - |  |

Supplementary Table 5. Sensitivity analysis of predicted RMR, poor functional outcome, ordinary mRS, and all-cause mortality

|  |  | Unadjusted | P value | Sex adjusted | P value | Multivariable adjusted¶ | P value | P for trend |
| --- | --- | --- | --- | --- | --- | --- | --- | --- |
|  |  | OR/HR (95%CI) |  | OR/HR (95%CI) |  | OR/HR (95%CI) |  |  |
| mRS ≥3 | Q 1 | Ref.§ (1) |  | Ref. (1) |  | Ref. (1) |  | P<0.001 |
|  | Q 2 | 0.73(0.63-0.84) | <0.0001 | 0.69(0.60-0.81) | <0.0001 | 0.69(0.58-0.83) | <0.0001 |  |
|  | Q 3 | 0.59(0.51-0.68) | <0.0001 | 0.54(0.45-0.64) | <0.0001 | 0.53(0.42-0.66) | <0.0001 |  |
|  | Q 4 | 0.47(0.41-0.55) | <0.0001 | 0.43(0.36-0.51) | <0.0001 | 0.42(0.32-0.56) | <0.0001 |  |
| Ordinary mRS | Q 1 | Ref. (1) |  | Ref. (1) |  | Ref. (1) |  | P<0.001 |
|  | Q 2 | 0.81(0.74-0.89) | <0.0001 | 0.77(0.70-0.85) | <0.0001 | 0.80(0.72-0.89) | <0.0001 |  |
|  | Q 3 | 0.73(0.67-0.80) | <0.0001 | 0.66(0.59-0.74) | <0.0001 | 0.69(0.61-0.79) | <0.0001 |  |
|  | Q 4 | 0.63(0.58-0.69) | <0.0001 | 0.57(0.51-0.63) | <0.0001 | 0.61(0.52-0.71) | <0.0001 |  |
| per 100 Kcal/d increment of RMR for mRS ≥3 | | 0.85(0.83-0.88) | <0.0001 | 0.82(0.80-0.85) | <0.0001 | 0.78(0.74-0.83) | <0.0001 |  |
| All-cause mortality | Q 1 | Ref. (1) |  | Ref. (1) |  | Ref. (1) |  | P<0.001 |
|  | Q 2 | 0.69(0.53-0.91) | 0.0081 | 0.59(0.44-0.79) | 0.0004 | 0.68(0.49-0.94) | 0.0209 |  |
|  | Q 3 | 0.64(0.48-0.84) | 0.0016 | 0.48(0.35-0.67) | <0.0001 | 0.59(0.40-0.87) | 0.0078 |  |
|  | Q 4 | 0.44(0.32-0.60) | <0.0001 | 0.32(0.22-0.46) | <0.0001 | 0.44(0.27-0.72) | 0.0010 |  |
| per 100 Kcal/d increment of RMR for all-cause mortality | | 0.84(0.79-0.88) | <0.0001 | 0.78(0.73-0.83) | <0.0001 | 0.79(0.72-0.88) | <0.0001 |  |

§Ref. indicates reference

¶Adjusted by sex, BMI, current smoking, hypertension, dyslipidemia, diabetes mellitus, prior stroke, prior atrial fibrillation, prior coronary heart disease, NIHSS, and mRS at admission, TOAST subtype, recombinant tissue plasminogen activator intravenous thrombolysis treatment, antithrombotic drugs, and lipid-lowering drug
